# Supplementary material for: Coincident molecular auxeticity and negative order parameter in a liquid crystal elastomer
Source: Nat Commun. 2018 Dec 4;9:5095. doi: 10.1038/s41467-018-07587-y (PMC6279820; doi:10.1038/s41467-018-07587-y)
Supplement: Supplementary file 1 — Supplementary Information [file 41467_2018_7587_MOESM1_ESM.pdf]

# Supplementary information for Coincident Molecular Auxeticity and Negative Order Parameter in a Liquid Crystal Elastomer

Mistry et. al.

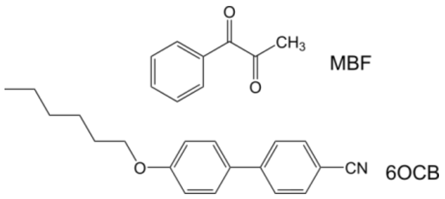


**Supplementary Figure 1 | Additional chemicals used in preparation of LCEs.** Chemical components used in the monomer state and washed from the LCE following polymerisation. Methyl benzoylformate (MBF) is a UV photoinitiator and 4’- hexyloxybiphenyl (6OCB) a liquid crystal required to give the monomer mixture the correct phase behaviour prior to polymerisation.

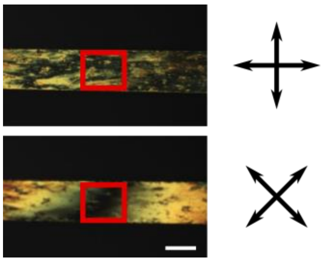

**Supplementary Figure 2 | Zero LC order in $xy$ plane at the onset of auxeticity.** Polarising microscopy images taken at of a sample tested at parameters **I** at the point prior to the emergence of auxetic behaviour ($\epsilon_x = 1.01$). The black appearance in the highlighted region in both photographs with the crossed polarisers indicates zero retardance and hence zero LC ordering within the image ($xy$) plane. Scale bar, 1 mm.

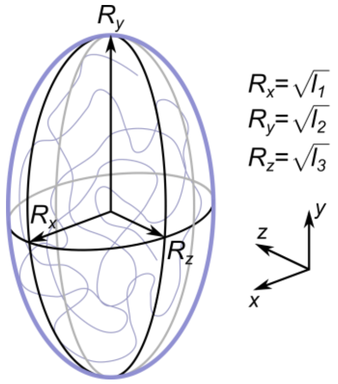


**Supplementary Figure 3 | Anisotropic polymer conformation of a LCE.** In general a LCE has a biaxially ellipsoidal polymer conformation which has different radii of gyration($R_x$, $R_y$, $R_z$) along each principle axis. The radii of gyration are related to the effective step lengths of the polymer backbone's anisotropic random walk as shown.

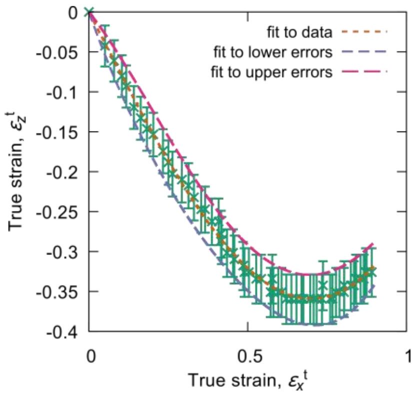


**Supplementary Figure 4 | Fitting to strain data for instantaneous PR calculation.** Plot of deformation behaviour for a sample tested according to conditions **I** (Table 1) plotted in terms of true strains, $\epsilon_i^t = \ln(\epsilon_i + 1)$. The instantaneous PR for the deformation in the $xz$ plane is determined from the negative gradient of 4$^{th}$ order polynomials fitted to the data shown. Fitting curves to the upper and lower bounds of the data allow estimates for the instantaneous PR to be calculated. Errors shown are measurement errors as described in the methods (n=1).
